# The National Dispensatory

**Published:** 1879-10

**Authors:** 


### BIBLIOGRAPHICAL.

THE NATIONAL DISPENSATORY containing the natural history, chemistry, Pharmacy, action and uses of medicines, including those recognized in the pharmacopœias of the United States, Great Britain and Germany, with numerous references to the French Codex. By Alfred Stille, M.D., L.L.D., Prof. of the theory and practice of medicine and of clinical midicine in the University of Pennsylvania, and John M. Maisch, Professor of Materia Medica and Botany in the Philadelphia college of Pharmacy. Second Edition, thoroughly revised, with numerous additions with two hundred and thirty nine illustrations. Philadelphia, Henry C. Lea. 1879.

This differs in some particulars from the United States Dispensatory: By Wood & Bache. An index of Therapeutics is an additional feature, while the rules of pharmacy so plainly given and beautifully illustrated by Wood & Bache, are entirely omitted in the new work before us. Many new remedies are included in this which were not known when the last revision of the United States Dispensatory was made, and in this particular the later work has advantages. It may be that early training biased the mind, but at any rate the impression exists that Dr. George B. Wood was the most correct and clear writer and teacher of pharmacy and acology in America.

